# Towards an international consensus on cross-border surrogacy: the role of the European Court of Human Rights?

**DOI:** 10.1093/medlaw/fwaf029

**Published:** 2025-08-13

**Authors:** Jakub Valc

**Affiliations:** Department of Legal Theory, Faculty of Law of Masaryk University, Brno 611 80, Czech Republic

**Keywords:** best interests of the child, cross-border surrogacy, international solution, margin of appreciation, right to private and family life, right to identity

## Abstract

The article examines ways to address the problems of cross-border surrogacy, based on existing initiatives to create an international legal framework and the case law of the European Court of Human Rights (ECtHR). I will first outline the problems associated with cross-border surrogacy and describe efforts to overcome these problems by adopting an international treaty or universally accepted standards in this area. Then I turn my attention to the nature of the right to private and family life and the general conditions for balancing this right against various public interests. The remainder of the article lies in a systematic analysis of the case law of the ECtHR on cross-border surrogacy, which has the potential to harmonize national approaches by establishing a minimum standard of human rights protection. I pay particular attention to the principle of the best interests of the child, the interpretation of which is a decisive factor and can thus serve as a general starting point for the resolution of human rights cases arising from cross-border surrogacy.

## I. INTRODUCTION

Surrogacy can generally be understood as the practice whereby one woman (the surrogate) carries a child for somebody else (the intended parents) by prior arrangement.^1^ Despite this general definition, surrogacy takes various forms. Based on the existence or absence of a genetic link between the surrogate and the child, a distinction can be made between traditional and gestational surrogacy.^2^ Another distinguishing feature is the altruistic or commercial nature of the procedure, where the decisive factor is not only the motivation of the surrogate^3^ and the related amount of payment or financial compensation, but also the (non)involvement of various for-profit entities in facilitating or carrying out surrogacy.^4^

Despite the diversity indicated, all forms of surrogacy as third-party reproduction^5^ can be considered as a specific way of establishing a family with the handover of the child after birth being pre-arranged; this is associated with a number of risks and problematic issues. Problems may also arise related to the diversity of social and legal conditions in different countries, leading to the development of reproductive tourism in the form of cross-border surrogacy based on international agreements on surrogacy.^6^ This is a type of cross-border reproductive care in which the intended parents cross national borders to access surrogacy that is illegal, unavailable, or inaccessible in their home country.^7^ Empirical data also support the various motives of intended parents for using a surrogate abroad. A study conducted in the UK in 2018 confirmed that a common reason respondents gave for using cross-border surrogacy was the more favourable legislation abroad.^8^ Another study in Australia in 2023 showed that the most common reasons were the unavailability of surrogates in the home jurisdiction, the possibility of professional facilitation and verification of the surrogate, the unenforceability of the surrogacy agreement, or the possibility of providing financial compensation to the surrogate.^9^

Whatever the motivation of the intended parents, cross-border surrogacy carries practical and legal risks for all the parties involved, including the child, who is the most vulnerable in the whole process. In addition to the general problem of exploitation,^10^ there are known controversial cases of the rejection of a disabled child by the intended parents.^11^ At the legal level, cross-border surrogacy involves not only the risk of the prosecution of the intended parents for circumvention of national prohibitions,^12^ but also potential problems regarding their return to their home country and continued cohabitation with the child if there are suspicions of non-compliance with legal conditions or even child trafficking.^13^ However, a general problem in cross-border surrogacy is the legal non-recognition of the parent–child relationship by the authorities of the home state of the intended parents, even if they have complied with foreign law.^14^

The aim of this article is to examine possible ways of preventing these problems or mitigating their effects by unifying or harmonizing rules at the international or regional level, including the related influence of the case law of the European Court of Human Rights (ECtHR) through the establishment of minimum standard of human rights protection in the development of national rules and in the decision-making of national authorities in cases of cross-border surrogacy. First, I focus on defining current initiatives seeking to find international consensus on the regulation of surrogacy or at least to harmonize rules for the legal recognition of parent–child relationships established in this way abroad. Then, I systematically analyse the case law of the ECtHR, whose legal opinions may partially compensate for the absence of binding international rules for resolving cases of cross-border surrogacy. I take into account the nature of the right to private and family life according to Article 8 of the European Convention on Human Rights (ECHR), including the special protection of the right to identity and the best interests of the child, which is relevant to all cases of cross-border surrogacy and whose interpretation significantly influences the requirements for decision-making in related matters.

## II. CURRENT EFFORTS TO FIND AN INTERNATIONAL SOLUTION TO CROSS-BORDER SURROGACY

A typical manifestation of ethically sensitive issues is the lack of international consensus on their regulation. Political decision-making in these areas is strongly culturally conditioned, making them literally national issues. This applies to the use of a wide range of modern methods of assisted reproduction. Although their gradual development and use to address infertility began as early as the second half of the last century,^15^ states are not uniform on whether, to whom, and under what conditions these health services can be provided. This is no different in the case of surrogacy. As with other methods of assisted reproduction, there is no binding international treaty that sets out a uniform and comprehensive legal framework for surrogacy at the global or regional level. This leads to a strong diversity of national approaches, which differ in whether, to whom, and under what conditions surrogacy is available. For the purposes of this article, it is sufficient to state that it is possible to encounter the absence of special legislation,^16^ a complete ban on surrogacy,^17^ or some sort of legalization of the procedure.^18^ Preventing cross-border surrogacy may then also consist of enacting access to surrogacy for state citizens only.^19^

However, cross-border surrogacy is a global issue that requires a global solution, including the related harmonization of national approaches. Such a solution could take various forms, ranging from a complete ban, through restrictions on certain forms, to the introduction of international standards of good practice.^20^ The demands for an absolute ban on surrogacy can be linked to the Casablanca Declaration, which has been supported by dozens of experts from all over the world who are members of various professions. The Declaration is a call for states to ban all forms of surrogacy on their territory, not to recognize the validity of surrogacy contracts, and to penalize any brokering or recruitment of surrogates. The authors of the Declaration are aware of the risk of the circumvention of national regulations, and therefore support the enforcement of these requirements at an international level, that is, by adopting an international treaty. Given that cross-border surrogacy usually takes place between states with different legal and cultural backgrounds, trying to reach such a broad consensus may be overly ambitious.^21^ One might, instead, expect the national implementation of these requirements, which would deepen the diversity of legal approaches and strengthen the use of cross-border surrogacy. One possible national response is to introduce severe penalties for the use of surrogacy by nationals not only in their home jurisdiction but also abroad, as Italy has recently done.^22^ The threat of repression can serve to prevent the circumvention of national rules. However, it is not a solution with a global impact and universal validity, which is the primary goal of the Declaration.

The relevant soft law instruments of the United Nations,^23^ the Council of Europe,^24^ and the European Union^25^ therefore seek to achieve less ambitious goals, such as a ban on, or strict control of, commercial surrogacy,^26^ a practice which is more controversial given the threat of exploitation and child trafficking.^27^ However, these are still non-binding documents, and some of them have implications only in the European legal area, so they may not produce the desired results because of the global nature of cross-border surrogacy. Another alternative to abolitionist approaches is standards of good practice, which encourage the introduction of procedural and other safeguards to protect children born from cross-border surrogacy. Among such standards are the Verona Principles, which consist of a total of 18 principles that take into account the vulnerability of such children.^28^ In the context of cross-border surrogacy, there is a significant emphasis on inter-state cooperation, in relation, inter alia, to the unification of rules on the recognition of the parent–child relationship and the related exercise of parental responsibility.^29^

This may be a compromise solution if no international consensus on surrogacy is found. The creation of such a legal framework would not have the effect of overriding the discretion of states to regulate surrogacy on their own territories, but it would only create a uniform and predictable procedural mechanism for the recognition of parent–child relationships that have been established in another state in accordance with local law and international norms. The Hague Conference on Private International Law (HCCH) has also been working on the creation of an international instrument on the legal recognition of parent–child relationships, inter alia, as a result of cross-border surrogacy. This is part of the so-called Surrogacy Project, which aims to establish universal rules for the recognition of parent–child relationships arising from international surrogacy agreements within the framework of a broadly conceived convention or in a separate protocol.^30^ The intention is to ensure greater predictability, certainty, and continuity in legal parenthood, in particular with regard to the protection of the rights and best interests of the child in accordance with the UN Convention on the Rights of the Child (UNCRC). However, a number of questions arise in relation to such an adoption of a protocol. It is not clear whether the protocol would apply only to the recognition of court decisions or would apply to other ways of determining parental status. In favour of the narrower concept is the possibility of applying uniform safeguards or standards,^31^ compliance with which would be a condition for recognition of parental status by a national court. In this context, the choice between an a priori and an a posteriori approach is to be considered.^32^ While the a priori approach requires prior cooperation between the authorities of the home states of the surrogate and the intended parents, the *a posteriori* approach does not require the involvement of the states concerned in the process before the conclusion of an international surrogacy agreement. The *a priori* approach represents a higher standard of predictability and protection for the persons involved, but requires the establishment and approval of a comprehensive mechanism of interstate cooperation and may be perceived negatively by some states as an implicit recognition of the legality of surrogacy. The a posteriori approach, on the other hand, requires some flexibility to be maintained in assessing compliance with the safeguards and decision-making, in relation to the best interests of the child and the continuity of the parent–child relationship that actually exists. Therefore, the question is not only the choice of one of the approaches, but also how the approach should be designed and implemented.^33^

Considering the international legal framework that already exists for the recognition of judicial decisions in the field of adoption,^34^ it seems that it would also be realistic to reach a consensus on the recognition of parent–child relationships arising from cross-border surrogacy.^35^ However, given the complex and sensitive ethical nature of surrogacy, adopting and ratifying a binding document will be a long process. The same is true of the European Commission’s proposal for a regulation on the recognition of parent–child relationships,^36^ according to which the applicable law should be the law of the state in which the woman giving birth is habitually resident or in which the child was born, even if it is the law of a third state. The aim is to unify the rules for national judicial and administrative authorities deciding on the recognition of parent–child relationships, including those arising from cross-border surrogacy. This should include the creation of a so-called European certificate that will be automatically recognized in Member States, thus ensuring legal certainty and predictability in decision-making.^37^ The regulation builds on European integration and will not affect the autonomy of Member States to regulate surrogacy, which increases the chances of its approval.^38^

Until the adoption of a binding legal instrument at a regional or international level, the recognition of foreign judgments or other legal documents in this area depends on national rules and their application. This does not mean that the actions of states and their authorities are not subject to any external constraints. There are international human rights obligations that can be directly affected by the non-recognition of the parent–child relationship, or by obstacles being placed on the development of such a relationship. The general requirements for the standard of protection of human rights then constitute common grounds that have a direct influence on the assessment of the legitimacy and proportionality of official procedures or decisions in these matters. These requirements do not derive directly from the text of international treaties, but come only from their interpretation by the competent authorities.

In the European context, the most influential role is played by the ECtHR, which has exclusive jurisdiction to rule on individual applications in relation to alleged violations of human rights enshrined in the ECHR. Proceedings before this judicial body result in a binding and enforceable judgment, which distinguishes this procedural mechanism from other international legal regimes for the protection of human rights, including the UNCRC. Although each ECtHR judgment is primarily binding on a particular Member State, its authority and relevance for the future adjudication of similar cases have a significant impact on the situation in other Member States.^39^ In other words, the case law of the ECtHR makes it possible to establish a minimum standard of human rights protection that allows national approaches in the treatment of cross-border surrogacy cases to be indirectly harmonized and allows the question of whether a certain interference with the human rights of the intended parents or the child is legitimate and proportionate to be addressed.^40^

## III. CROSS-BORDER SURROGACY AS A HUMAN RIGHTS CONFLICT UNDER ARTICLE 8 ECHR: A BALANCING PROCESS

### A. ECHR: General principles and categorization of protected rights

When interpreting and applying the ECHR, it is necessary to take into account the fundamental principles on which this international treaty relies. One of the main objectives of the ECHR is effectiveness in maintaining and realizing human rights, which means that the ECHR does not guarantee theoretical or illusory rights, but rights that have practical implications and effects.^41^ This is linked to the principle of subsidiarity, which reflects the fact that the protection of human rights is the responsibility of national authorities, while the ECtHR merely verifies compliance with the obligations arising under the ECHR.^42^ When assessing an individual complaint, the ECtHR first examines whether the facts fall within the scope of any of the guaranteed human rights, taking into account the evolutionary interpretation or conception of the ECHR as a living instrument.^43^ The ECtHR then assesses whether the right in question has actually been interfered with and whether such interference was legitimate and reasonably justified.^44^ In this step, not only the principle of proportionality plays a key role but also the application of the margin of appreciation doctrine, which allows national preferences to be reflected in the resolution of sensitive ethical issues on which there is no consensus among Member States.^45^ However, the way in which complaints are assessed also takes into account the typology of guaranteed rights, particularly in relation to their absolute or non-absolute nature, respectively, their variously limited derogability or non-derogability.^46^

### B. Scope of the right to private and family life

No international treaty, including the ECHR, grants a right to undertake surrogacy in one’s own country or abroad. However, relationships that have been established in this way are protected because they fall within the scope of the right to private and family life under Article 8 ECHR. This is a broad category that cannot be exhaustively defined, as it is shaped on a case-by-case basis and changes with social and technological progress.^47^ It encompasses various aspects of an individual’s psychological and physiological life, which may be intrapersonal or interpersonal in nature, and which often interact with each other. This is evidenced by the scope of private life, which refers to various manifestations of physical and psychological integrity that have both personal and social dimensions. The protection of private life covers both the autonomous development of an individual’s personality and self-perception (personal identity)^48^ and the establishment, maintenance, and development of various relationships with others and the outside world (social identity).^49^ Because of this, it is necessary to distinguish the nature of family life as an autonomous concept that is associated only with close personal ties.^50^ The assessment of the nature of these ties depends not on their legal recognition but on their actual existence and the way they operate. In other words, protection applies to family life de facto, not just de jure.^51^ In this respect, various circumstances are relevant, which include not only cohabitation but also the nature, intensity, and duration of the relationship in question.^52^ The existence of family life within the meaning of Article 8 ECHR cannot therefore be presumed, as it depends on a case-by-case assessment, which also applies to the relationship between the legal parent and the child. Indeed, the legal parent does not have to be the social parent at the same time, which is relevant for the assessment of the mutual emotional bond and its intensity. This is related to the purpose of protecting family life, which is to ensure the undisturbed cohabitation and normal development of family relationships, but only if they actually exist.^53^

### C. The composition of conflicting human rights and public interests

These principles are also applicable in the field of cross-border surrogacy when the maintenance or development of the parent–child relationship is impeded by practical or legal obstacles that may arise from the actions, decisions, or inactions of state authorities. The related effects may then manifest themselves in both family and private life. For family life, this will be the case if the personal contact and emotional bond between the child and the intended parents is temporarily or permanently disrupted. Experience has shown that the temporary separation of a child can be caused by a refusal to issue a travel document on the grounds that it is not properly documented that the entire process was carried out in accordance with the law. Without a valid visa or travel document, the child cannot travel, while the intended parents must return to their home country if they have no legal basis for obtaining or extending a residence permit.^54^ The permanent removal of the child from the care of the intended parents can then be based on a court decision if it is proved that there has been a violation of the law, for example, because of the lack of a biological link between the child and at least one of the intended parents. In other words, the establishment of a parent–child relationship is the result of an unlawful act.^55^ The possibility of developing a family life will thus be not only temporarily limited, but completely prevented. A special category is represented by cases in which, although the intended parents can return to their home country with the child, the national authorities do not officially recognize the parental status of one or both of them.^56^ Such an official procedure does not have to affect specific aspects of family life, as it may not lead to a disruption of the de facto cohabitation between the child and the intended parents. However, the non-recognition of the parent–child relationship may have implications for the child’s private life, particularly the protection of his or her identity and the associated possibility of full integration into society.^57^

It can therefore be concluded that the use of surrogacy abroad creates many problems that are relevant in the context of interference with the right to private and family life of the intended parents and the child. However, such interference is not necessarily impermissible, as it may be justified by the protection of public interests. The identification and importance of those interests then depend on the individual circumstances. The decision not to issue travel documents and to prevent the child from entering the territory of the intended parents’ home state is aimed at preventing human trafficking if there are reasonable doubts about the intended parents’ intentions or the legality of the procedure.^58^ The permanent removal of a child from the care of the intended parents may be rationally justified if the intended parents’ actions constitute a violation of the law, typically in the sense of an unauthorized change of personal status or the falsification of an official document. The aim is to bring to an end the unlawful situation and to provide a suitable environment for the upbringing of the child.^59^ On the other hand, failure to meet the requirements for the use of surrogacy abroad is not a ground for non-recognition of the parent–child relationship in the intended parents’ home state. Such a procedure is usually based on the application of the public policy reservation, a legal rule that allows a foreign decision not to be recognized if such recognition would be incompatible with the basic ideas of a statutory regulation and the principles of justice expressed therein, which must be insisted upon.^60^ This will be the case in states that explicitly prohibit surrogacy or consider it to be contrary to legally recognized ways of starting a family and acquiring parental status, and in which there is an unquestioning application of the traditional principle of *mater semper certa est*.^61^

### D. The margin of appreciation and its limitations

From a human rights perspective, all court cases of cross-border surrogacy create a conflict between individual and public interests that must be balanced against each other in order to achieve a fair balance between them.^62^ This means that even the right to private and family life is not absolute in nature, as the protection of this right may give way to the protection of the rights of other persons or public interests, provided that the requirement of proportionality is respected.^63^ The requirements for such balancing then depend on the nature of the issue at stake and the particular aspects of private or family life that are to be disregarded in favour of another individual or public interest. In this respect, Member States have a wide margin of appreciation when they regulate ethically sensitive issues on which there is no consensus.^64^ As already explained, the legal regulation of surrogacy is not uniform within the member states of the Council of Europe.^65^ Member States, therefore, generally have a wide margin of appreciation in regulating surrogacy.^66^ Nevertheless, the scope of the margin of appreciation varies, as cross-border surrogacy may affect the right to private and family life of the intended parents and the child in different ways. In this context, the ECtHR distinguishes between the peripheral areas of the right in question, on the one hand, and the core of that right, in terms of its integral components, on the other. In relation to the latter category, it concludes that the scope of the Member States’ margin of appreciation is a priori limited or subject to strict scrutiny.^67^ In other words, interference with one of the integral components of a given right leads to its increased protection and the limitation of the margin of appreciation in the protection of other values. Such integral components include the right to identity, which may affect multiple partial rights and interests of a child born to a surrogate abroad.^68^

In the case law of the ECtHR, the right to the identity of the child resulting from cross-border surrogacy has been addressed in the context of cases of the non-recognition of a foreign birth certificate that was intended to form the grounds for the registration of the intended parents’ parental status in the national civil registry.^69^ Its interpretation has thus focused on the negative effects of the denial of legal recognition of the parent–child relationship on the legal status of the child and the child’s related ability to exercise inheritance and other rights. However, future cases concerning the impact on other components or manifestations of the child’s identity can be expected.^70^ The solutions to these cases may not be identical, but they will undoubtedly reflect the general premise of the nature and level of protection of the right to identity, which should be approached in a holistic manner in accordance with international standards.^71^

Interference with identity is not the only argument that can add weight to the imaginary scales in favour of a child. Another important argument has become the best interests of the child, even though this is not enshrined in the ECHR at all. Its normative significance derives from Article 3(1) UNCRC, which provides that the best interests of the child must be a primary consideration in any action concerning a child. It is the cornerstone and value base of the entire system for the international protection of the rights of the child,^72^ and can be understood in several ways. First, it can be seen as a substantive right that is a primary consideration in balancing the various interests in matters concerning the child. Secondly, it can function as a procedural rule or a requirement to adopt specific procedural safeguards or measures to protect the child as a vulnerable person. Thirdly, it can serve as a default interpretation principle, meaning that it determines which of several possible interpretations of the child’s right is to be preferred.^73^

The above-mentioned concepts of the best interests of the child are also applied when interpreting the right to private and family life under Article 8 ECHR, which applies to a wide range of human rights holders without explicitly reflecting the special position of the child.^74^ In this context, the best interests of the child serve, in particular, as one of the important factors in resolving a conflict between the child’s right to private and family life and a public interest. The principle also functions as a methodological tool for selecting the most appropriate of several possible interpretations of a given right, thus becoming a standard part of the ECtHR’s decision-making.^75^

Nevertheless, the best interests of the child are not an absolute principle or value, as they are only a primary consideration in the evaluation of various official actions affecting the child.^76^ In other words, the best interests of the child have a stronger position than other values, reflecting the immaturity of the child and the vulnerability associated with this. Nevertheless, the best interests of the child should not operate as an axiom that always prevails regardless of the circumstances of the case and the nature of the conflicting public interests. In this context, the Committee on the Rights of the Child stresses that the practical application of the principle of the best interests of the child in resolving a human rights conflict requires the maintenance of a degree of flexibility to allow for the importance of the sphere of the integrity of the child concerned, on the one hand, and the composition of the conflicting principles, on the other.^77^ This means that the best interests of the child may be the object of balancing and may also, in exceptional cases, give way to the protection of other interests or values.^78^ The nature of the issue and the circumstances of the particular case are determinative. This is no different in the case of cross-border surrogacy, which affects various aspects of a child’s private and family life. The recommendation of the Special Rapporteur on the sale and sexual exploitation of children, then, explicitly places the best interests of the child first among the factors to be considered in assessing the parent–child relationships thus established.^79^ However, this is a form of soft law that is not binding and merely provides a general starting point for decision-making. The consideration of individual circumstances is reflected only in the case law of the ECtHR, which is case-based in nature and gives the concept of the best interests of the child a realistic form.

## IV. IMPLEMENTATION OF BALANCING CRITERIA IN RESOLVING CASES OF CROSS-BORDER SURROGACY

The balancing process always depends on the individual circumstances of the case, which vary enormously. The common feature is the best interests of the child, which underpins all cases of cross-border surrogacy. However, the interpretation of this principle can be equally problematic, as it is a subjective assessment that can only be reconstructed on the basis of an analysis of particular decisions and the reasoning used in them.^80^ Any attempt to formulate general conclusions in this direction is further complicated by the diversity of cases in the field of cross-border surrogacy, but this can be partially overcome by categorizing the cases according to common factual features. For this purpose, the following analysis will be divided into three categories of cases that have emerged in the case law of the ECtHR.

### A. Temporary separation of the child from the intended parents

The temporary separation of the child from the intended parents is related to the case of a Belgian couple who used surrogacy in Ukraine. Although the intended parents were listed on the birth certificate, the Belgian Embassy in Kiev refused to issue a travel document for the child because it did not have enough information to verify the intended parents’ parentage. The Belgian court came to the same conclusion until the biological paternity of the intended father had been sufficiently proved. However, as a result of the expiry of the intended parents’ residence permits, the child had to remain in Ukraine for several months in the care of a nanny, with only regular visits from the intended parents, which they considered to be a violation of their right to private and family life under Article 8 ECHR.^81^

According to the ECtHR, it was not disputed that the applicants had cared for the child from birth and continued to do so after the obstacle to the child’s removal had been lifted. In the meantime, they had taken a genuine interest in him and, despite the complications, had visited him regularly. A family life within the meaning of Article 8 ECHR has therefore been established between them. The enforced separation must have been emotionally difficult for both the applicants and the child, since sustained contact with parents is essential for any child’s healthy development. However, this right had to be balanced against the important public interests in monitoring compliance with the law and combating human trafficking, which also related to the individual protection of the surrogate and the child.^82^ Despite the wide margin of appreciation, the measures taken must be proportionate, and this depends on the individual circumstances. In that connection, the ECtHR emphasized that the separation of the child from the applicants had lasted only a few months, particularly because the proceedings before the national court were conducted under a simplified procedure. Moreover, the break in contact was the result of the applicants’ misconduct, even though they had consulted a Belgian and a Ukrainian lawyer.^83^

The ECtHR’s reasoning shows that combating child trafficking in such cases may be not only in the public interest, but also in the individual interests of the surrogate and the child. This creates a complicated situation in which the child has an interest in maintaining contact with the applicants as well as in being protected as a potential victim of trafficking. There is therefore a problem in determining what is in the best interests of the child in the circumstances. The ECtHR did not give a clear answer to this question here, as it did not address the relevance of these different interests to the decision. Nor did it address the level of risk of child trafficking and alternative ways of ensuring the child’s protection, such as official supervision after return to the home country of the intended parents, preventing further travel with the child until the end of court proceedings, etc.^84^ In other words, the ECtHR did not transparently reflect the relevant facts in the legal assessment. On the other hand, the ECtHR correctly concluded that the child’s interest in maintaining contact with the applicants could also have been undermined by the short periods of separation associated with their regular visits. For a newborn child, continuity of personal contact with parents is generally more intense, inter alia, in the context of the establishment of an emotional bond. The applicants’ failure to prove a biological link could not then be construed against the child, who had no influence on their behaviour. Therefore, the ECtHR should have considered the violations of the rights of the intended parents and the child separately, although their arguments were naturally overlapping. Moreover, the ECtHR did not address the fact that the disruption of family ties can affect the establishment of a child’s identity as an important aspect of his or her private life, the protection of which limits the margin of appreciation.^85^ It can be concluded that the best interests of the child were not clearly identified in the judgment, and their specific role in the balancing exercise remained unclear.

### B. Permanent removal of the child from the intended parents’ care

The interpretation of the best interests of the child also proved problematic in another cross-border surrogacy case, which led to the removal of the child from the care of Mrs Donatina Paradiso (first applicant) and Mr Giovanni Campanelli (second applicant) and to his being given up for adoption to another couple. This was the case of a married Italian couple who used surrogacy in Russia. After the birth of the child, they obtained an internationally recognized birth certificate in which they were listed as the child’s parents. However, the Italian authorities refused to recognize the parent–child relationship because of the absence of a biological link between the child and the second applicant, which also resulted in a criminal prosecution for unauthorized change of personal status and falsification of an official document. The child, therefore, had no legal parents and was placed in a children’s home for adoption. The national courts justified this course of action on the basis of a legitimate desire to put an end to the unlawful situation. The courts then challenged the conclusions of the applicants’ psychological report regarding the negative effects of the removal of the child on his development, basing their challenge on his age, the short time he had spent with the applicants, and the imminent search for a new family.^86^

The Chamber of the ECtHR took a different view on the interpretation of the best interests of the child. The Chamber concluded that the non-recognition of the birth certificate could only exclude the legal relationship between the child and the applicants, not the creation of a family life that existed between them. Similarly, the intervention of the Italian authorities may have had an impact on the private life of the second applicant, who sought recognition of the parent–child relationship. In considering the case, the Chamber took into account both the public interest in ending the unlawful situation and the protection of the abandoned child. This was similar reasoning to that in *D and others v Belgium*, but the breach of the rules had been proved. In the Chamber’s view, the action taken by the national authorities was contrary to the best interests of the child, since removal from care is an exceptional measure to be taken when a child is in imminent danger.^87^ The national authorities did not sufficiently consider the circumstances of the child’s cohabitation with the applicants, and that consideration was necessary to determine the best interests of the child and to strike a fair balance.^88^

However, the Chamber’s decision was overturned by the Grand Chamber of the ECtHR, to which the case was referred under Article 43 ECHR. The Grand Chamber agreed with the conclusion that family life can, under certain conditions, be based only on personal ties and not on legal and biological ties.^89^ In accordance with earlier case law, the Grand Chamber considered that the relevant criteria for the assessment of the case were the role of the applicants in relation to the child, the quality of their ties, and the length of their cohabitation. It concluded that the applicants had demonstrably assumed a parental role, which they had exercised responsibly. Their cohabitation with the child had lasted several months, which is a relatively short period of time.^90^ The parental role and the quality of emotional ties were not enough to establish the existence of family life. However, the applicants’ private life was affected, since they had long aspired to become parents, and this fell within the scope of the right in question.^91^ In balancing the right to private life, the Grand Chamber relied in particular on a wide margin of appreciation. That scope was not limited by the protection of the child’s identity, since the application could not have been lodged on his behalf and nor was the subject of the proceedings the recognition of his biological link to the second applicant. It was therefore not only the short period of cohabitation and the absence of a biological link that weighed against the applicants, but also the breach of the legal rules. Their right was outweighed by the public interest in bringing an end to the unlawful situation, the importance of which was supported by the strict prohibition of surrogacy in Italian law. Even the best interests of the child did not justify a contrary conclusion. The Grand Chamber recalled that the principle in question is a primary consideration that must be taken into account with regard to the real impact on the child, even if he is not the applicant.^92^ In interpreting the best interests of the child, the Grand Chamber concluded that the domestic courts were faced with a certain dilemma as to whether they should de facto approve the applicants’ unlawful conduct or immediately provide another suitable environment for the child’s upbringing. The Grand Chamber concluded that the preference for the second option did not exceed the margin of appreciation and could be in the best interests of the child. That conclusion was in contradiction to the psychological report, but the Grand Chamber considered that it was legitimate for a national court with the appropriate specialization to challenge the report.^93^

The Grand Chamber’s judgment can be considered controversial, as evidenced by the fact that several of the judges filed concurring and dissenting opinions. One of the main issues of concern was the interpretation of the best interests of the child. The authors of the concurring opinion referred to commercial surrogacy and the corresponding conduct of the applicants as human trafficking.^94^ This supported the conclusion that removing the child from the applicants’ illegal custody was in the child’s best interests. The opposite approach, which I consider to be correct, is evident in the joint dissenting opinion, whose authors point out the illogical reasoning of the Grand Chamber, which recognizes the assumption of the parental role and the creation of an emotional bond while affirming the abandonment of the child. Such a conclusion is based on an interpretation of the domestic law that is inconsistent with its purpose and divorced from reality.^95^ Moreover, it contradicts the true essence of the best interests of the child, which is to pursue the well-being of the child.^96^ This generally means that it is preferable to maintain permanent contact with the parents, unless this contact is harmful to the child.^97^ In other words, from the child’s perspective, what matters is not how the relationship was established but that it actually exists and that maintaining it is positive for the child’s development. The protection of the parent–child relationship is all the stronger in cases of removal from care and forced adoption. In such a situation, the best interests of the child become not only a primary but also the paramount consideration for decision-making.^98^

However, the Grand Chamber dealt with the best interests of the child in a minimalist manner without emphasizing the child’s real needs. These needs were also clearly identified in the psychological report, which was challenged by the national courts on the basis of expert literature without the standard request for a review report.^99^ In contrast to the case of *D and others v Belgium*, the Grand Chamber thus clearly defined what it sees as the best interests of the child. Specifically, the Grand Chamber understood the best interests of the child to be the quick ending of the unlawful situation, which can generally be understood more as a public interest that should be balanced against the individual rights and interests of the child.^100^ The Grand Chamber simply reversed this logic with no evidence of any negative effects of continued cohabitation with the applicants on the child’s development, and no evidence or confirmation of human trafficking.

### C. Non-recognition of foreign birth certificates and intended parents’ parental status

Cases of the non-recognition of a parent–child relationship resulting from cross-border surrogacy are related to the application of the public policy exception in states that prohibit or criminalize surrogacy. In these cases, it is important who the intended parents are and whether or not they have a biological link to the child, as this can bring the right to identity into play.^101^ One of the first cases of this kind was the ECtHR judgment in *Mennesson v France*.^102^ A surrogate gave birth to twins for a heterosexual married couple using sperm from Mr Mennesson (the first applicant) and eggs from a donor (that is, a person different from both the surrogate and Mrs Mennesson as the second applicant). Prior to the birth of the daughters (the third and fourth applicants), the California Supreme Court recognized the first and second applicants as legal parents. However, the French consulate initially refused to enter the data in the national register because there had been a breach of the law, and the civil court subsequently annulled and deleted the entry that had been made in the meantime,^103^ which the applicants challenged as a violation of Article 8 ECHR.^104^

The ECtHR assumed that there had been a legal basis for the national authorities’ action and that the first and second applicants must have been aware of the registration problems. Only the question of the proportionality of the interference with the applicants’ rights became decisive. The ECtHR examined the case both from the point of view of the family life of all the applicants and from the point of view of the private life of only the third and fourth applicants. The question had become manifest in the scope of the margin of appreciation and resolution of the human rights conflict. As regards family life, the ECtHR did not dispute that the failure to recognize the parent–child relationship would create obstacles in a number of situations in the lives of the third and fourth applicants, who were not French nationals. Nevertheless, the applicants were able to settle and live together in France like other families in a comparable situation, without facing any real threat or fear of separation.^105^ Although this was an interference with the right to family life, it was legitimate and proportionate. However, the ECtHR concluded that the non-recognition of the parent–child relationship created uncertainty as to the third and fourth applicants’ acquisition of nationality from their biological father. This had the effect of making integration into French society more difficult and excluded the possibility of legal succession from the first applicant, since the third and fourth applicants were de jure strangers to him. The ECtHR decided that this was not a fair balance because the margin of appreciation was limited by the protection of the identity and best interests of the third and fourth applicants, who were in the position of children. The ECtHR thus clearly decided that the best interests of the child were contravened by the non-recognition of the parent–child relationship on a biological basis, which is what was actively sought to be established.^106^ The conduct of the national authorities was therefore considered to be a violation of the right to a private life of the third and fourth applicants.^107^

However, the ECtHR’s conclusions were primarily concerned with the legal recognition of the relationship with the biological father. The question remained unanswered as to whether the Member State was also obliged to recognize a parent–child relationship with an intended mother who had no biological link to the child. The answer to this question was only provided by the ECtHR’s opinion of 2019,^108^ which followed the judgment in *Mennesson v France* after the national authorities had only entered the first applicant’s parental status in the civil registry. This had led to the reopening of the original proceedings, including the referral of the case to the ECtHR for an opinion and answers to several questions. Because of the nature of the opinion and its connection with the particular case, the ECtHR focused only on the questions of whether the failure to register the intended mother (originally the second applicant) was a violation of Article 8 ECHR and, if so, whether the desired goal could also be achieved by the adoption of the child by the intended mother as the wife of the biological father. According to the ECtHR, the persistent inconsistency of national approaches leaves a wide margin of appreciation as to how to discourage the use of surrogacy domestically or internationally. However, such measures have an impact on the child by creating uncertainty about his or her position in society in various ways. The inability to acquire the nationality of the intended mother may adversely affect the child’s right of residence, his or her capacity to inherit, or the ability and obligation of the intended mother to care for him or her in the event of the death of the second parent.^109^ Generally speaking, the failure to recognize the bond between parent and child can jeopardize the stability of the child’s upbringing and prevent the child from being fully integrated into society. This touches not only on identity,^110^ but also on the best interests of the child, which can have different dimensions here. This interpretation may strengthen measures against child abuse or the child’s interest in having access to information about his or her genetic or biological origin (namely, the identity of the surrogate or egg donor). Nevertheless, the ECtHR concluded that an absolute and generally applied obstacle to the recognition of a parent–child relationship is incompatible with the essence of the best interests of the child, which must be assessed on a case-by-case basis.^111^

According to the ECtHR, it is not necessary to achieve this goal by recognizing a foreign birth certificate. What is essential is that the legal uncertainty regarding the relationship between the child and the intended mother be removed as soon as possible. For this purpose, Member States may make use of the various legal rules or procedural mechanisms available in the legal order, including adoption proceedings.^112^ The method chosen must make it possible to remove the legal uncertainty quickly and effectively, as soon as the relationship between the child and the intended mother becomes a reality.^113^ However, the ECtHR has suggested that the use of adoption can be complicated if it is legally available only to heterosexual married couples.^114^ Intended parents may also be unmarried couples, homosexual couples, or single people. This was demonstrated in *DB and others v Switzerland*,^115^ a case in which a man from a same-sex couple was not allowed to adopt the child of his registered partner, with whom he had used a surrogate abroad. Swiss law prohibited both surrogacy and adoption by a person in a registered partnership. Therefore, the intended parent with no biological link to the child could not achieve any form of recognition of the parent–child relationship. The ECtHR considered the state’s measure incompatible with the best interests of the child, which it identified with the need to be able to legally identify the persons responsible for his or her upbringing. The best interests of the child, therefore, cannot be conditioned by the particular sexual orientation or behaviour of the intended parents, even if this involves circumventing national rules.^116^ In other words, responsibility for circumventing national rules cannot be transferred to the child.

It can be concluded that, in cases of the non-recognition of the parent–child relationship, the identity and best interests of the child constitute clearly identifiable and applicable arguments for resolving the related human rights conflict. Nevertheless, these arguments may not necessarily lead to success in the case from the applicants’ perspective. The application assessment is largely dependent on the individual circumstances, including the applicant’s behaviour and the true impact on the child’s development. There is no impermissible interference with the right to private and family life if alternative ways of removing uncertainty about the parent–child relationship are not used or are frustrated as a result of the applicants’ behaviour.^117^ Even in such a situation, the right to identity and the best interests of the child cannot be disregarded. However, the state may take the best interests into account by making an extraordinary decision to grant the child citizenship and to place the child in the care of one or both of the applicants through permanent foster care.^118^ Such a solution contributes to the integration of the child into society and to the stability of the environment for his or her upbringing, even though it may not correspond to what the applicants have formally sought. For the same reason, efforts to obtain multiple nationalities from the biological parents may not be successful if the child lives with the parents in a state in which they are all nationals, with the possibility of full integration.^119^

## V. CONCLUSION

All previous attempts to address the problem of cross-border surrogacy by harmonizing national approaches have so far been unsuccessful. An international consensus on the recognition of parent–child relationships arising from cross-border surrogacy could solve some problems. However, a number of unresolved issues remain, and a binding legal framework cannot be expected soon. Attention is therefore drawn to the case law of the ECtHR, which, at the regional level, goes some way to harmonizing national approaches through the establishment of a minimum standard of protection of the rights of the intended parents and the child. The common starting point of these cases is the best interests of the child, which allows for a limitation on an otherwise wide margin of appreciation in balancing the rights and public interests involved. However, the best interests of the child may be interpreted in different ways, and even the protection of this principle is not absolute. Analysis has shown that this principle can be used to argue for both the preservation of the parent–child relationship and the temporary or permanent separation of the child from the intended parents. The use of this principle by the ECtHR is therefore casuistic and not completely predictable. A gradual strengthening of legal certainty can be observed in cases of the legal recognition of parent–child relationships, where the principle of the best interests of the child is accompanied by the right to identity, which enjoys increased protection and is not limited to biological ties or conditioned by a particular sexual orientation of the intended parent. The combination of these principles thus becomes a decisive weight on the imaginary scales. It creates a standard that partially overcomes the unpredictability and inconsistency in the use of the margin of appreciation doctrine, which has been subject to criticism. The previous ECtHR judgments therefore establish a positive obligation on states to recognize, in some way, the parent–child relationship resulting from cross-border surrogacy where the best interests of the child and the protection of the child’s identity require it. However, the question is how these conclusions will be applied to specific family models in the future and whether they will lead to a liberalization of the rules on access to surrogacy in Member States whose restrictive approach can be effectively circumvented in this way. Nevertheless, I am of the opinion that legal certainty requires the creation of a binding international or regional legal framework, at least on the issue of the legal recognition of the parent–child relationship, even though the adoption of such a framework will be time-consuming and will probably require the maintenance of a certain degree of flexibility in deciding individual cases.

Conflict of interest. None declared.

## Footnotes

1 E Brodeală and MH Peter-Spiess, ‘Surrogacy and Same-Sex Parenthood before the European Court of Human Rights: Reflections in Light of Cases against Switzerland’ (2022) 32 Swiss Review of International and European Law 397.

2 L Bracken, ‘Assessing the Best Interests of the Child in Cases of Cross-border Surrogacy: Inconsistency in the Strasbourg Approach?’ (2017) 39 Journal of Social Welfare and Family Law 368.

3 Payments or other compensation do not necessarily preclude altruism or the surrogate’s motivation to help someone else. See K Horsey, ‘The Future of Surrogacy: A Review of Current Global Trends and National Landscapes’ (2024) 48 Reproductive BioMedicine Online <https://www.rbmojournal.com/article/S1472-6483(23)00863-5/fulltext> accessed 12 January 2025.

4 The altruistic form is typically associated only with the reimbursement of reasonable or itemized costs. See E Kneebone, K Beilby and K Hammarberg, ‘Experiences of Surrogates and Intended Parents of Surrogacy Arrangements: A Systematic Review’ (2022) 45 Reproductive BioMedicine Online <https://www.rbmojournal.com/article/S1472-6483(22)00416-3/fulltext> accessed 12 January 2025.

5 According to the American Society for Reproductive Medicine, this term ‘refers to involving someone other than the individual or couple that plans to raise the child (intended parent[s]) in the process of reproduction. This includes using donated eggs, sperm, or embryos and gestational-carrier arrangements, in which the pregnancy is carried by someone other than the intended parent(s)’. See ASRM, ‘Third-Party Reproduction: A Guide for Patients’ (ASRM 2018) <https://www.reproductivefacts.org/globalassets/_rf/news-and-publications/bookletsfact-sheets/english-pdf/third-party_reproduction_booklet_web.pdf> accessed 13 January 2025.

6 The international character of such a contract can be understood as the result of the habitual residence of the surrogate and the intended parents being in different states, together with the intention to subsequently take the child to the home country of the intended parents. See HCCH, ‘Parentage/Surrogacy Experts’ Group: Final Report, “The Feasibility of One or More Private International Law Instruments on Legal Parentage’ (HCCH, 1 November 2022) <https://assets.hcch.net/docs/6d8eeb81-ef67-4b21-be42-f7261d0cfa52.pdf> accessed 13 January 2025.

7 K Hammarberg, M Stafford-Bell and S Everingham, ‘Intended Parents’ Motivations and Information and Support Needs when Seeking Extraterritorial Compensated Surrogacy’ (2015) 31 Reproductive BioMedicine Online <https://www.rbmojournal.com/article/S1472-6483(15)00416-2/fulltext> accessed 11 January 2025.

8 V Jadva, H Prosser and N Gamble, ‘Cross-border and Domestic Surrogacy in the UK Context: An Exploration of Practical and Legal Decision-making’ (2021) 24 Human Fertility 93.

9 E Kneebone and others, ‘Australian Intended Parents’ Decision-making and Characteristics and Outcomes of Surrogacy Arrangements Completed in Australia and Overseas’ (2023) 26 Human Fertility 1448.

10 Exploitation of surrogates can be emotional or economic in nature. See P Gerber and K O’Byrne, *Surrogacy, Law and Human Rights* (Routledge 2016) 47.

11 There is a well-known case of Baby Gammy, in which an Australian couple refused to take custody of a child with Down syndrome. See A Whittaker, *International Surrogacy as Disruptive Industry in Southeast Asia* (Rutgers University Press 2019) 25–26.

12
*Mennesson v France* App no 65192/11 (ECtHR, 26 September 2014); *Labassee v France* App no 65941/11 (ECtHR, 26 September 2014).

13
*D and others v Belgium* App no 29176/13 (ECtHR, 8 July 2014); *Paradiso and Campanelli v Italy* (2017) 65 EHRR 22.

14 The ECtHR has addressed this issue in a number of decisions, which will be discussed later, including the pivotal decision in *Mennesson v France* (n 12); The basis for such a procedure is the desire to protect the fundamental values of the legal system of the given state, including the related protection of the rights and interests of the child and the surrogate. See C Mayer, ‘Ordre public und Anerkennung der rechtlichen Elternschaft in internationalen Leihmutterschaftsfällen’ [2014] 78 RabelsZ 551.

15 RA Frankel, J Merkison and AH Decherney, ‘ART: Right to Reproduce’ in J Schenker and others (eds), *Hot Topics in Human Reproduction: Ethics, Law and Society* (Springer 2023) 69.

16 On the situation in European countries, see CDBIO, ‘Surrogacy: Addendum to the Replies to Questionnaire on Access to Medically Assisted Procreation (MAP), on Right to Know About Their Origin for Children Born After MAP’ (Council of Europe, 2 April 2024) <https://rm.coe.int/inf-2022-13-addendum-february-2024-e-2759-8499-0985-v-1/1680b0601c> accessed 24 January 2025.

17 Examples of countries in this category are Germany, Austria, Switzerland, France, Italy and China. See K Trimming and P Beaumont, *International Surrogacy Arrangements: Legal Regulation at the International Level* (Hart Publishing 2013) 463.

18 National legislation varies, inter alia, in terms of for whom (heterosexual couples, same-sex couples, single people) and in what form (gestational/traditional, commercial/altruistic) surrogacy is available, and how the parental status of intended parents is determined. See Center for Genetics and Society, ‘Surrogacy Regulation by Country’ (*Surrogacy360*) <https://surrogacy360.org/considering-surrogacy/current-law/> accessed 14 January 2025; This applies also to the USA, where there is no federal regulation of surrogacy. See Creative Family Connections, ‘The US Surrogacy Law Map’ (Creative Family Connections) <https://creativefamilyconnections.com/us-surrogacy-law-map/> accessed 14 January 2025.

19 Countries in this category include Russia, India or Thailand. See Center for Genetics and Society (n 18).

20 This also requires taking into account the realities of the global market. See C Fenton-Glynn and JM Scherpe, ‘Surrogacy in a Globalised World: Comparative Analysis and Thoughts on Regulation’ in JM Scherpe, C Fenton-Glynn and T Kaan (eds), *Eastern and Western Perspectives on Surrogacy* (Intersentia 2019) 591–92.

21 Some have pointed to the risk that a global ban on surrogacy would shift international surrogacy arrangements to the black market, which would only deepen exploitation problems. See Trimming and Beaumont (n 17) 442.

22 European Institute of Bioethics, ‘Italy Recognizes Surrogacy as a “Universal Crime”: Symbolic Measure or First Step towards a Global Ban?’ (European Institute of Bioethics, 29 October 2024) <https://www.ieb-eib.org/en/news/early-life/surrogacy/italy-recognizes-surrogacy-as-a-universal-crime-symbolic-measure-or-first-step-towards-a-global-ban-2300.html> accessed 15 January 2025.

23 Special Rapporteur on the Sale and Sexual Exploitation of Children, ‘Report on Safeguards for the Protection of the Rights of Children Born from Surrogacy Arrangements’ (OHCHR, 15 July 2019) <https://documents.un.org/doc/undoc/gen/g18/007/71/pdf/g1800771.pdf> accessed 24 January 2025.

24 P De Sutter, ‘Children’s Rights Related to Surrogacy’ (Parliamentary Assembly of the Council of Europe, 23 September 2016) <https://assembly.coe.int/nw/xml/XRef/Xref-XML2HTML-en.asp?fileid=23015&lang=en> accessed 24 January 2025.

25 European Parliament resolution of 17 December 2015 on the Annual Report on Human Rights and Democracy in the World 2014 and the European Union’s policy on the matter (2015/2229(INI)).

26 This is related to the prohibition of commercialization of the human body and its parts under Article 21 of the European Convention on Human Rights and Biomedicine, which does not exclude the reimbursement of reasonable costs. See Council of Europe, ‘Explanatory Report to the Convention on Human Rights and Biomedicine’ (Council of Europe, 4 April 1997) <https://rm.coe.int/16800ccde5> accessed 24 January 2025.

27 The exploitation of surrogates is a form of human trafficking under Directive (EU) 2024/1712 of the European Parliament and of the Council of 13 June 2024, amending Directive 2011/36/EU on preventing and combating trafficking in human beings and protecting its victims.

28 International Social Service, ‘Principles for the Protection of the Rights of the Child Born through Surrogacy (Verona Principles)’ (International Social Service, 25 February 2021) <https://iss-ssi.org/storage/2023/03/VeronaPrinciples_25February2021-1.pdf> accessed 13 January 2025.

29 ibid.

30 A separate protocol is intended to increase the chances of such a convention being adopted. See HCCH (n 6) 25–26.

31 For a list of the possible safeguards, see ibid 32–34.

32 Another option is a combined approach based on an a posteriori approach with an opt-in/opt-out choice of an a priori approach. See ibid 29.

33 ibid 28–29.

34 Hague Convention on Protection of Children and Co-operation in Respect of Intercountry Adoption.

35 Fenton-Glynn and Scherpe (n 20) 573; Trimming and Beaumont (n 17) 442.

36 Proposal for a Council Regulation on jurisdiction, applicable law, recognition of decisions and acceptance of authentic instruments in matters of parenthood and on the creation of a European Certificate of Parenthood 2022/0402(CNS).

37 Alina Tryfonidou, ‘Cross-Border Legal Recognition of Parenthood in the EU’ (European Parliament, 2023) <https://www.europarl.europa.eu/RegData/etudes/STUD/2023/746632/IPOL_STU(2023)746632_EN.pdf> accessed 24 January 2025.

38 This does not preclude some modifications, including the need to take into account related HCCH activities.

39 A Seibert-Fohr and ME Villiger, ‘Current Challenges in European Multilevel Human Rights Protection’ in A Seibert-Fohr and ME Villiger (eds), *Judgments of the European Court of Human Rights—Effects and Implementation* (Nomos 2014) 13.

40 J Gerards, ‘Margin of Appreciation and Incrementalism in the Case Law of the European Court of Human Rights’ (2018) 18 Human Rights Law Review 495.

41
*Airey v Ireland* (1979) 2 EHRR 305, para 24.

42 J Gerards, *General Principles of the European Convention on Human Rights* (CUP 2019) 5.

43 This means that the ECHR must be interpreted in the light of present-day conditions. See S Wheatley, ‘Interpreting the ECHR in Light of the Increasingly High Standards Being Required by Human Rights: Insights from Social Ontology’ (2024) 24 Human Rights Law Review 1.

44 ibid 18.

45 Depending on the scope, a distinction can be made between ‘narrow margin’, ‘certain margin’, and ‘wide margin’. However, this doctrine is subject to frequent criticism due to the ambiguity of its application, the undermining of the universality of human rights, or the questioning of the standard of their protection. See A Legg, *The Margin of Appreciation in International Human Rights Law: Deference and Proportionality* (OUP 2012) 1.

46 The doctrine distinguishes between the following categories: (i) Absolutely absolute ‘rights or non-derogable rights’ (eg Article 3), (ii) Absolute rights that are not ‘notstandsfest’ (eg Article 5), (iii) Non-absolute or derogable rights with express, specific limitation clauses (eg Article 2), (iv) Non-absolute or derogable rights with express, general limitation clauses (eg Article 8), (v) Non-absolute or derogable rights without express limitation clauses, which allow for implied or inherent limitations (eg Article 6), (vi) Other non-absolute or derogable rights (eg Article 12). See Gerards (n 42) 19–30.

47
*Niemietz v Germany* (1993) 16 EHRR 97; *Pretty v United Kingdom* App no 2346/02 (ECtHR, 29 July 2002); *Peck v United Kingdom* App no 44647/98 (ECtHR, 28 April 2003).

48
*Von Hannover v Germany* (2006) 43 EHRR 7; *A.-M.V. v Finland* App no 53251/13 (ECtHR, 23 March 2017).

49
*Bărbulescu v Romania* App no 61496/08 (ECtHR, 5 September 2017); *Mikulić v Croatia* App no 53176/99 (ECtHR, 4 September 2002).

50
*Paradiso and Campanelli v Italy* (n 13) para 140.

51
*Marckx v Belgium* (1979) 2 EHRR 330; *Johnston and others v Ireland* (1987) 9 EHRR 203.

52
*X, Y and Z v the United Kingdom* App no 21830/93 (ECtHR, 22 April 1997).

53
*Marckx v Belgium* (n 51) para 31.

54
*D and others v Belgium* (n 13) para 14.

55
*Paradiso and Campanelli v Italy* (n 13) para 189.

56 This is dealt with in a series of judgments of the ECtHR. See eg, *Mennesson v France* (n 12); *D v France* App no 11288/18 (ECtHR, 16 October 2020); *Valdís Fjölnisdóttir and others v Iceland* App no 71552/17 (ECtHR, 18 May 2021); *DB and others v Switzerland* App no 58817/15 and 58252/15 (ECtHR, 22 November 2022); *C v Italy* App no 47196/21 (ECtHR, 31 August 2023); *AM v Norway* App no 30254/18 (ECtHR, 24 March 2022); *KK v Denmark* App no 25212/21 (ECtHR, 6 Dezember 2022).

57
*Mennesson v France* (n 12) para 96; *Labassee v France* (n 12).

58
*D and others v Belgium* (n 13) para 59.

59
*Paradiso and Campanelli v Italy* (n 13) para 189.

60 Mayer (n 14) 571.

61 The mother is always certain and is considered to be the woman who gave birth to the child. See D Gruenbaum, ‘Foreign Surrogate Motherhood: mater semper certa erat’ (2012) 60 American Journal of Comparative Law 475.

62
*Odièvre v France* (2004) 38 EHRR 43; *Evans v UK* (2006) 43 EHRR 21; *Dickson v UK* App no 44362/04 (ECtHR, 4 December 2007).

63 This explicitly follows from art 8(2) ECHR.

64
*X, Y and Z v the United Kingdom* (n 52); *Fretté v France* (2004) 38 EHRR 21.

65 Limiting the margin of appreciation requires broad interstate consensus. Increasing tendencies towards regulatory convergence alone are not enough. See *SH and others v Austria* App no 57813/00 (ECtHR, 3 November 2011); *Costa and Pavan v Italy* App no 54270/10 (ECtHR, 11 February 2013).

66
*H v United Kingdom* App no 32185/20 (ECtHR, 31 May 2022).

67
*Jäggi v Switzerland* (2008) 47 EHRR 30.

68 The right to identity is also related to the right to birth registration, name, nationality, and information on genetic or biological origin. In addition, it indirectly affects the right to the child’s development, the preservation of family ties, and the continuity of ethnic and cultural background. See Trimming and Beaumont (n 17) 108.

69 A Mulligan, ‘Identity Rights and Sensitive Ethical Questions: The European Convention on Human Rights and the Regulation of Surrogacy Arrangements’ (2018) 26 Medical Law Review 449.

70 This is the impact on the child’s right to know his or her origins or the identity of the surrogate as the genetic or biological mother. The interpretation of this right has also been addressed by the ECtHR in the context of anonymous gamete donation. See *Gauvin-Fournis and Silliau v France*, App no 21424/16 and 45728/177 (ECtHR, 7 September 2023).

71 International Social Service (n 28).

72 R Ruggiero, ‘The Best Interest of the Child’ in Z Vaghri and others (eds), *Monitoring State Compliance with the UN Convention on the Rights of the Child* (Springer 2022) 22.

73 Committee on the Rights of the Children, ‘General Comment No. 14 (2013) on the Right of the Child to Have his or her Best Interests Taken as a Primary Consideration (Art. 3, Para. 1)’ (*United Nations*, 29 May 2013) <https://www2.ohchr.org/english/bodies/crc/docs/gc/crc_c_gc_14_eng.pdf> accessed 24 January 2025.

74 J Collinson, ‘Making the Best Interests of the Child a Substantive Human Right at the Centre of National Level Expulsion Decisions’ (2020) 38 Netherlands Quarterly of Human Rights 169.

75 European Union Agency for Fundamental Rights and Council of Europe, *Handbook on European Law Relating to the Rights of the Child* (Publications Office of the European Union 2022) 90.

76 In the 1959 Declaration of the Rights of the Child, the best interests of the child were even considered a ‘paramount consideration’. See Ruggiero (n 72) 22.

77 ibid 27.

78 UNHCR, *UNHCR Guidelines on Determining the Best Interests of the Child* (UNHCR 2008) 76.

79 The recommendation literally refers to the best interests of the child as the ‘paramount consideration’. See Special Rapporteur (n 23) 19.

80 De Sutter (n 24) 8–9.

81
*D and others v Belgium* (n 13) paras 1–18.

82
*Rantsev v Cyprus and Russia* (2010) 51 EHRR 1.

83
*D and others v Belgium* (n 13) para 60.

84 The best interests of the child require that all reasonably available means be used to ensure that the separation of the child from his or her parents is as short as possible. See U Kilkelly, ‘Protecting Children’s Rights under the ECHR: The Role of Positive Obligations’ (2020) 61 Northern Ireland Legal Quarterly 245.

85 D McGoldrick, ‘A Defence of the Margin of Appreciation and an Argument for its Application by the Human Rights Committee’ (2015) 65 International & Comparative Law Quarterly 21.

86 For more details on the facts, see *Paradiso and Campanelli v Italy* (n 13) paras 8–56.

87
*Scozzari and Giunta v Italy* (2002) 35 EHRR 12; *Neulinger and Shuruk v Switzerland* (2012) 54 EHRR 31.

88
*Paradiso and Campanelli v Italy* (n 13) paras 81–86.

89
*Moretti and Benedetti v Italy* App no 16318/07 (ECtHR, 27 April 2010); *Kopf and Liberda v Austria* App no 1598/06 (ECtHR, 17 January 2012).

90 Longevity of cohabitation as one of the factors influencing the assessment of family life is generally calculated in years rather than months. See *Wagner and JMWL* App no 76240/01 (ECtHR, 28 June 2007); *Nazarenko v Russia* (2019) 69 EHRR 6.

91
*Evans v UK* (n 62) para 71; *Dickson v UK* (n 62) para 66; *SH and others v Austria* (n 65) para 82.

92
*X v Latvia* (2014) 59 EHRR 3.

93
*Paradiso and Campanelli v Italy* (n 13) para 212.

94 ibid (joint concurring opinion) para 6; This conclusion does not reflect the difficulties in defining commercial surrogacy, in which financial compensation to a surrogate is not always considered human trafficking. See Horsey (n 3).

95 ibid (joint dissenting opinion) para 8.

96 UNHCR (n 78) 14.

97
*Neulinger and Shuruk v Switzerland* (n 87) para 101; *R and H v the United Kingdom* (2012) 54 EHRR 2.

98
*Johansen v Norway* (1997) 23 EHRR 33; *Kearns v France* (2010) 50 EHRR 33; *YC v the United Kingdom* (2012) 55 EHRR 33.

99 For criticism of this procedure, see also Bracken (n 2) 376.

100 Ruggiero (n 72) 25–26.

101 In *Paradiso and Campanelli v Italy*, the biological link was not present, so it was only a matter of interpretation of the best interests of the child. The ECtHR has traditionally understood identity in relation to the genetic or biological link between a child and a parent. See Mulligan (n 69) 470–71.

102
*Mennesson v France* (n 12).

103 This distinguishes this case from the otherwise similar case of Mr and Mrs. Labassee, who sought recognition of the existence of a parent–child relationship on the basis of de facto status, not through direct recognition of a foreign birth certificate. See *Labassee v France* (n 12).

104 For more details on the facts, see *Mennesson v France* (n 12) paras 6–28.

105
*Chavdarov v Bulgaria* App no 3465/03 (ECtHR, 21 December 2010).

106 However, there may be cases where the best interests of the child conflict with the legal recognition of the biological parent’s relationship and the custody of the child. See *AL v France* App no 13344/20 (ECtHR, 07 April 2022).

107
*Mennesson v France* (n 12) paras 96–101.

108 Advisory opinion concerning the recognition in domestic law of a legal parent–child relationship between a child born through a gestational surrogacy arrangement abroad and the intended mother. Request no P16-2018-001 (ECtHR, 10 April 2019).

109 On the effects of non-recognition of the parent–child relationship on the identity of the child, see *KK v Denmark* (n 56).

110 Personal identity includes how a person perceives themselves and how others perceive them and behave towards them. See D Layder, *Social and Personal Identity* (Sage 2004) 7.

111 Advisory opinion (n 108) para 42; In essence, the ECtHR confirmed that the recognition of a parent–child relationship with the intended mother is also derived from the marriage to the biological father. See A Margaria, ‘Parenthood and Cross-Border Surrogacy: What Is “New”? The ECtHR’s First Advisory Opinion’ (2020) 28 Medical Law Review 412.

112 Advisory opinion (n 108) para 54; *D v France* (n 56) para 85.

113 What is essential is the existence of a real relationship between the intended mother and the child, which weakens the previous importance of genetic or biological parenthood. See Brodeală and Peter-Spiess (n 1) 411.

114 Advisory opinion (n 108) para 57.

115
*DB and others v Switzerland* (n 56).

116 See also *Foulon and Bouvet v France*, App no 9063/14 and 10410/14 (ECtHR, 21 October 2016); ibid.

117 This does not just refer to not using the possibility of adoption, but also covers not requesting the partial registration of data from a foreign birth certificate. The behaviour of the applicants may also consist of the fact that they get divorced during the adoption procedure, which makes it impossible to complete that procedure in accordance with the law. See *Bonzano and others v Italy* App no 10810/20 (ECtHR, 14 December 2021); *Valdís Fjölnisdóttir and others v Iceland* (n 56); *Modanese and others v Italy* App no 59054/19 (ECtHR, 30 May 2023).

118
*Valdís Fjölnisdóttir and others v Iceland* (n 56) para 71.

119
*SH v Poland* App no 56846/15 and 56849/15 (ECtHR, 16 November 2021).

